# Supplementary material for: Spatial Distribution of Immune Cells in Primary and Recurrent Glioblastoma: A Small Case Study
Source: Cancers (Basel). 2023 Jun 20;15(12):3256. doi: 10.3390/cancers15123256 (PMC10297152; doi:10.3390/cancers15123256)
Supplement: Supplementary file 1 [file cancers-15-03256-s001.zip › cancers-2425676-supplementary.pdf]

Supplemental Data

Target
**β-Catenin**
**CD8**
**B7-H3**
**CD4**
**FOXP3**
**Mouse IgG1**
**CD68**
**PTEN**
**IgG Rabbit Isotype**
**CD14**
**S6**
**GZMB**
**Ki-67**
**β-2-macroglobulin**
**Histone H3**
**AKT**
**CD3**
**CD66B**
**VISTA**
**PD-1**
**pSTAT3**
**CD44**
**STAT3**
**CD56**
**PDL-1**
**CD45**
**p-AKT**
**HLA-DR**
**CD20**
**pan-Cytokeratin**
**CD45RO**
**Bcl-2**
**B7-H4 VTCN1**
**STING TMEM173**
**IDO-1**
**CD11c**
**ICOS CD278**
**OX40L CD252 TXGP1**
**CD163**

Table S1: List of antigens analysed in this study

A

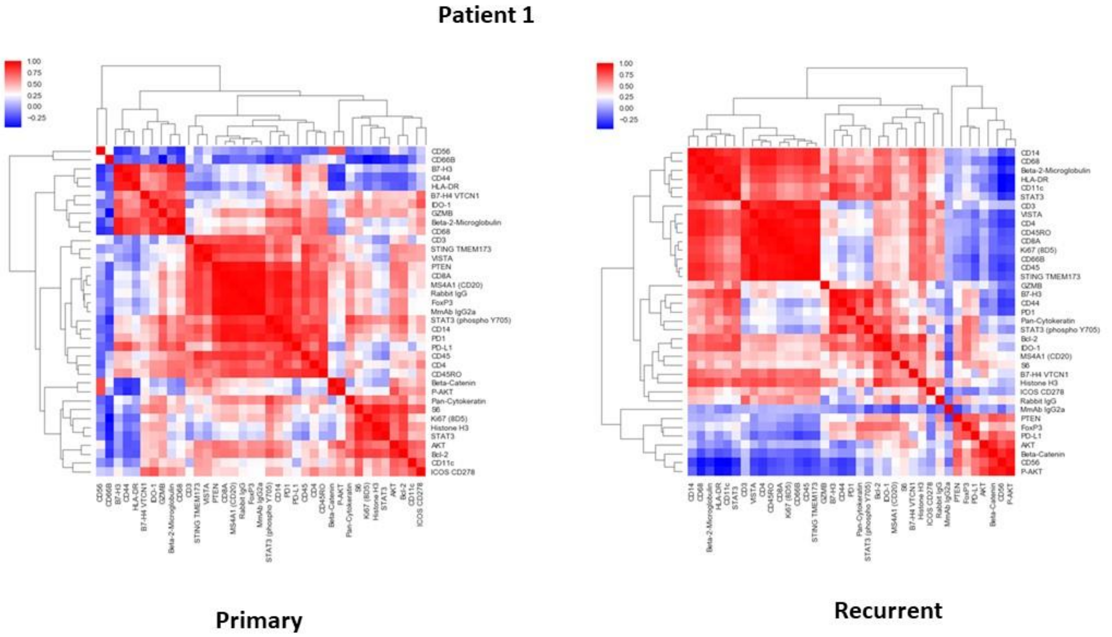

Figure S1A : Loussouarn et al.

B

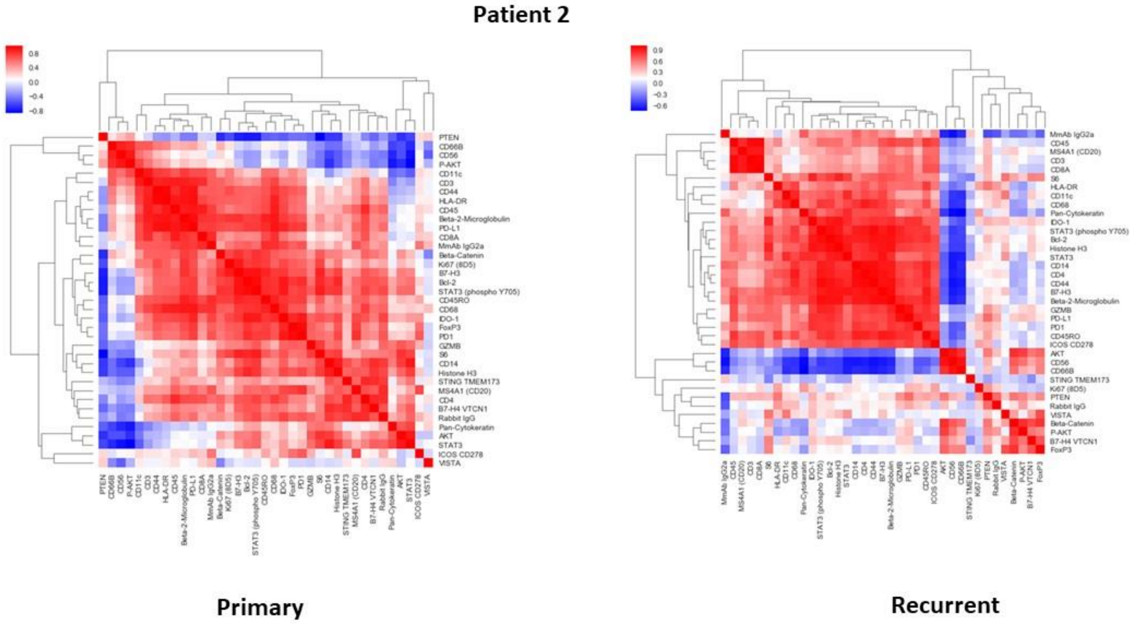

Figure S1B : Loussouarn et al.

C

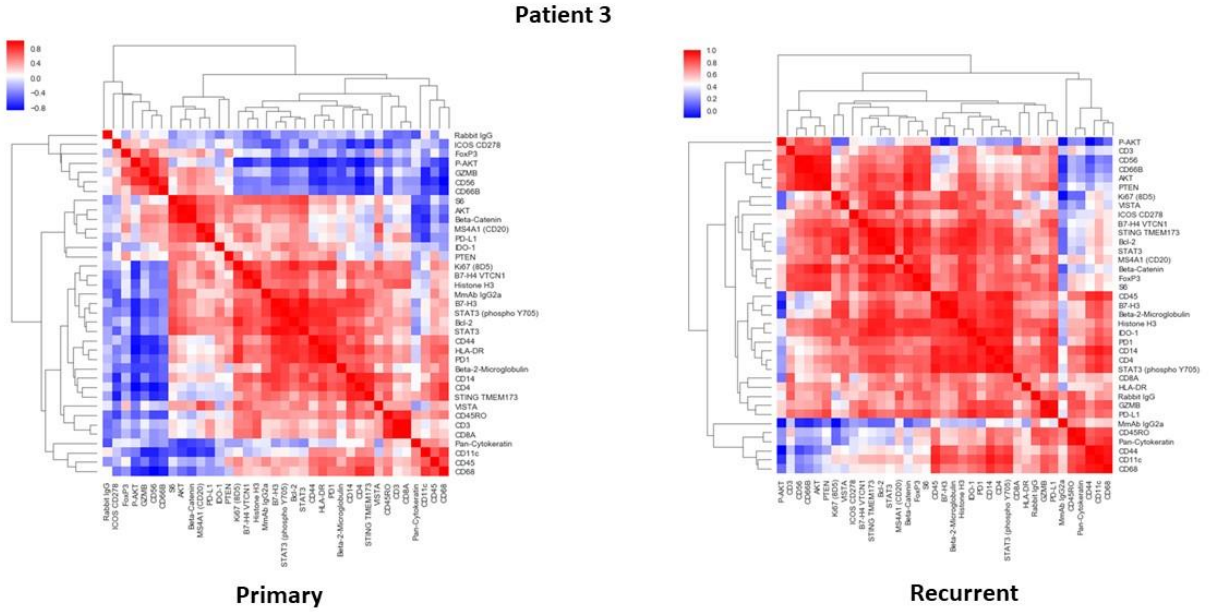

Figure S1C : Loussouarn et al.

D

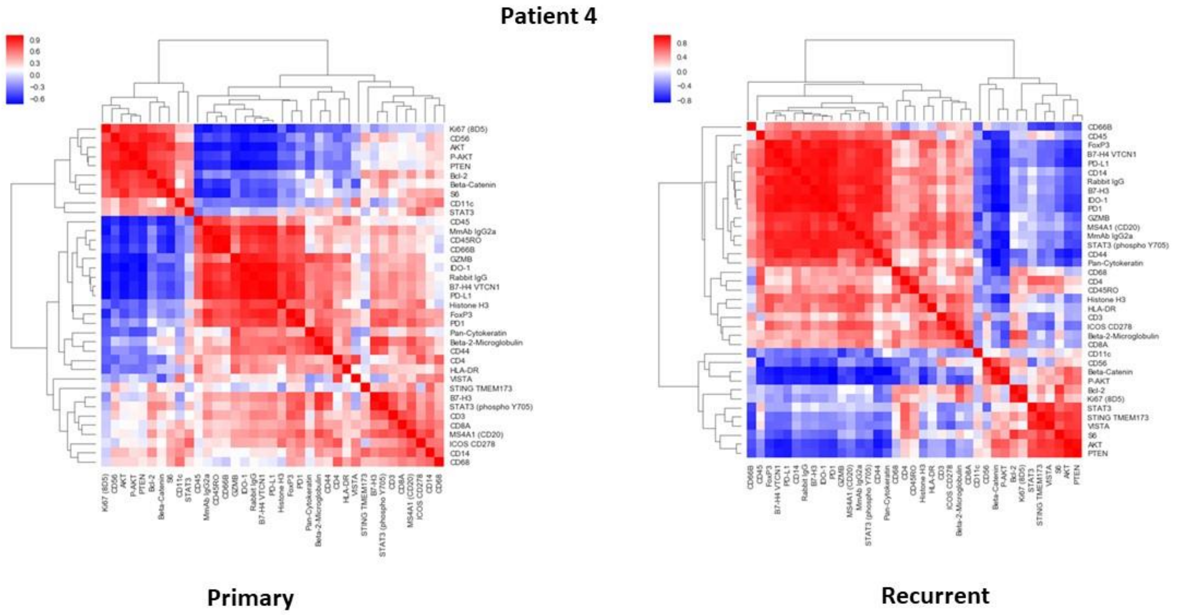

Figure S1D: Loussouarn et al.

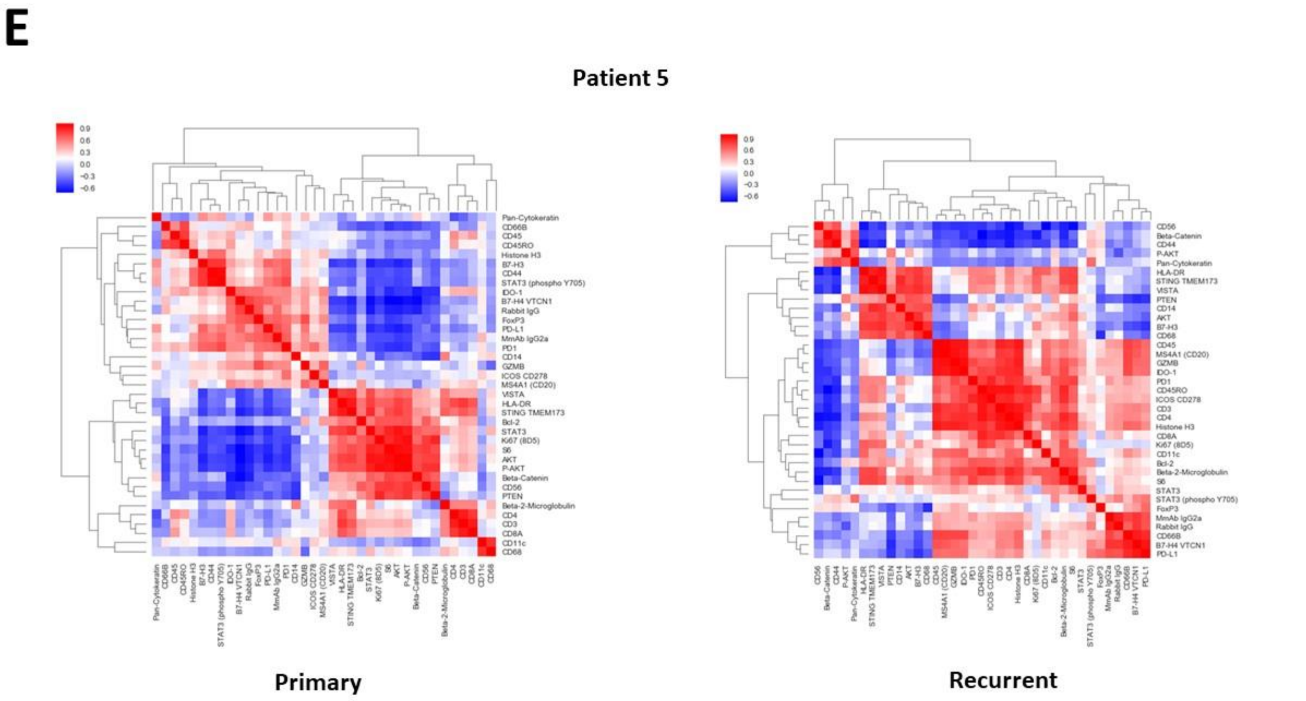

Figure S1E: Loussouarn et al.

Figure S1 (A-E): correlation matrix between the DSP markers in the 5 primary and recurrent tumors.

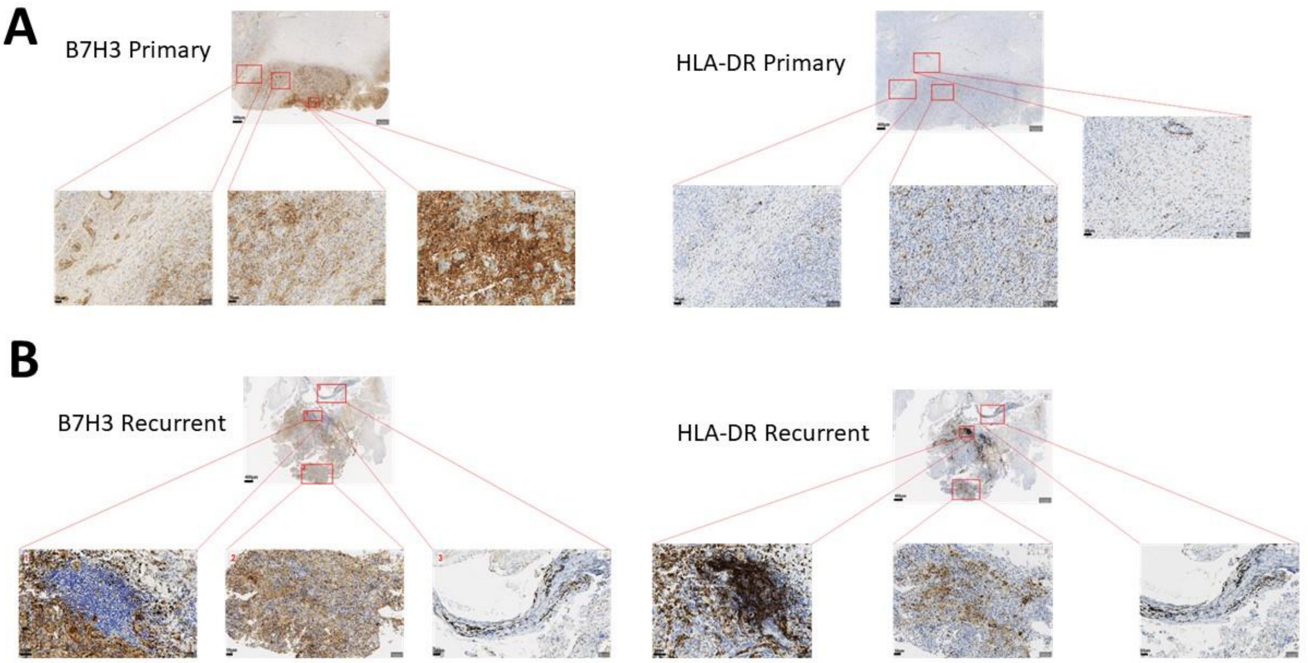

Figure S2: Loussouarn et al.

Figure S2: Immuno-histochemistry images of tumor labelling of HLADR and B7H3 in representative primary GBM (A) and recurrent GBM (B).
